# Contributions to Chinese fauna of Torrenticolidae Piersig, 1902 (Acari, Hydrachnidia), with the description of three new species

**DOI:** 10.3897/zookeys.955.52584

**Published:** 2020-08-05

**Authors:** Xin-Yao Gu, Lan Jia, Dao-Chao Jin, Jian-Jun Guo

**Affiliations:** 1 Institute of Entomology, Guizhou University, Guizhou Provincial Key Laboratory for Plant Pest Management of the Mountainous Region, the Scientific Observing and Experimental Station of Crop Pest in Guiyang, Ministry of Agriculture, China, Guiyang 550025, China Guizhou University Guiyang China

**Keywords:** China, morphology, running waters, taxonomy, torrenticolid mites, water mites

## Abstract

Five species of torrenticolid mites (Acari, Hydrachnidia), collected in the Anzihe and Qingliangfeng national nature reserves, R. P. China, are identified. Three species are described as new to science: *Torrenticola pseudosiamis* Gu & Guo, **sp. nov.**, *T. anziensis* Gu & Guo, **sp. nov.**, and *Monatractides sichuanensis* Gu & Guo, **sp. nov.** The other two species, *M. macrocorpis* Gu & Guo, 2019, *M. xiaoxiensis* Gu & Guo, 2019, are newly reported from Zhejiang Province. Descriptions and illustrations of these species are included.

## Introduction

China is rich in ecological diversity and types of water bodies, which suggests that a rich species diversity of water mites is expected in the Chinese fauna. In the number of species, the family Torrenticolidae is one of the largest groups of water mites. Until now, the total number of torrenticolid mites is about 600 worldwide, but only 38 species are known from China, including the three new species added in this paper (Gu et al. 2019a, 2020a, b, c; Gu and Guo 2019). This means that the Chinese torrenticolid mites fauna is largely unknown, and the identification of Chinese torrenticolid species will be a focus in the near future.

In this paper, five species of Torrenticolidae are identified from two national nature reserves in China, Anzihe National Nature Reserve in Sichuan Province and Qingliangfeng National Nature Reserve in Zhejiang Province. Three of these species are new to science: *Torrenticola pseudosiamis* Gu & Guo, sp. nov., *T. anziensis* Gu & Guo, sp. nov., and *Monatractides sichuanensis* Gu & Guo, sp. nov. The other two are newly reported from Zhejiang Province: *M. macrocorpis* Gu & Guo, 2019 and *M. xiaoxiensis* Gu & Guo, 2019. These five species are described and illustrated here.

## Material and methods

Water mites were collected, preserved, cleaned, and mounted following the usual methods (Jin 1997; Ding et al. 2019).

The following abbreviations are used (Jin 1997; Wiles 1997; Goldschmidt 2007; Zhang 2018): aL = apical length; Ap = anal pore; bs = basal segment of chelicera; Cx-I–Cx-IV = coxae I–IV; dL = dorsal length; I-L-1–6, etc. = first leg’s segment 1–6, etc.; L = length; mL = medial length; Gf = genital field; GUGC = Institute of Entomology, Guizhou University, Guiyang, China; P-1–5 = palp segment 1–5; vL = ventral length; W = width. The chaetotaxy used follows Jin (1997): *A_2_* = postantennal glandularia; *D_1_*–*D_4_* = dorsoglandularia 1–4; *E_2_*, *E_4_* = epimeroglandularia 2, 4; *L_1_*–*L_4_* = lateroglandularia 1–4; *O_2_* = postocularia; *V_1_*–*V_4_* = ventroglandularia 1–4; 4+1 = five plates: four anterior platelets and a single large dorsal plate.

All measurements of palp and legs are of the dorsal margin, given in micrometers (μm) and following Goldschmidt (2007). All the specimens examined are kept in GUGC (no. ZJ-TO-20180701–ZJ-TO-20180709, SC-TO-20160701–SC-TO-20160704).

## Taxonomy

### Class Arachnida Lamarck, 1801

#### Order Trombidiformes Reuter, 1909

**Family Torrenticolidae Piersig, 1902**

**Subfamily Torrenticolinae Piersig, 1902**

**Genus *Torrenticola* Piersig, 1896**

##### Torrenticola pseudosiamis

Taxon classificationAnimaliaTrombidiformesTorrenticolidae

Gu & Guo sp. nov.

E9AF6221-6BC9-539B-B90F-4B7297DF7346

http://zoobank.org/C8F4222B-E74D-4A0E-BE01-80751961123F

Figures 1 , 2

###### Material examined.

***Holotype***: ♀ (ZJ-TO-20180701), Qingliangfeng National Nature Reserve, Lin’an, Zhejiang Province, P. R. China (30°6'44"N, 118°53'36"E, 940 m a.s.l.), collected by Xinyao Gu, 31-VII-2018. ***Paratype***: 2 ♀♀ (ZJ-TO-20180702, ZJ-TO-20180703), same data as holotype.

###### Diagnosis.

Idiosoma elliptical; dorsal plate 4+1; infracapitular bay U-shaped; *E_4_* at the same level as the 6^th^ pair of acetabula; gnathosoma with a short rostrum, the lateral view of gnathosoma regular triangle-like.

###### Description.

**Figure 1.**
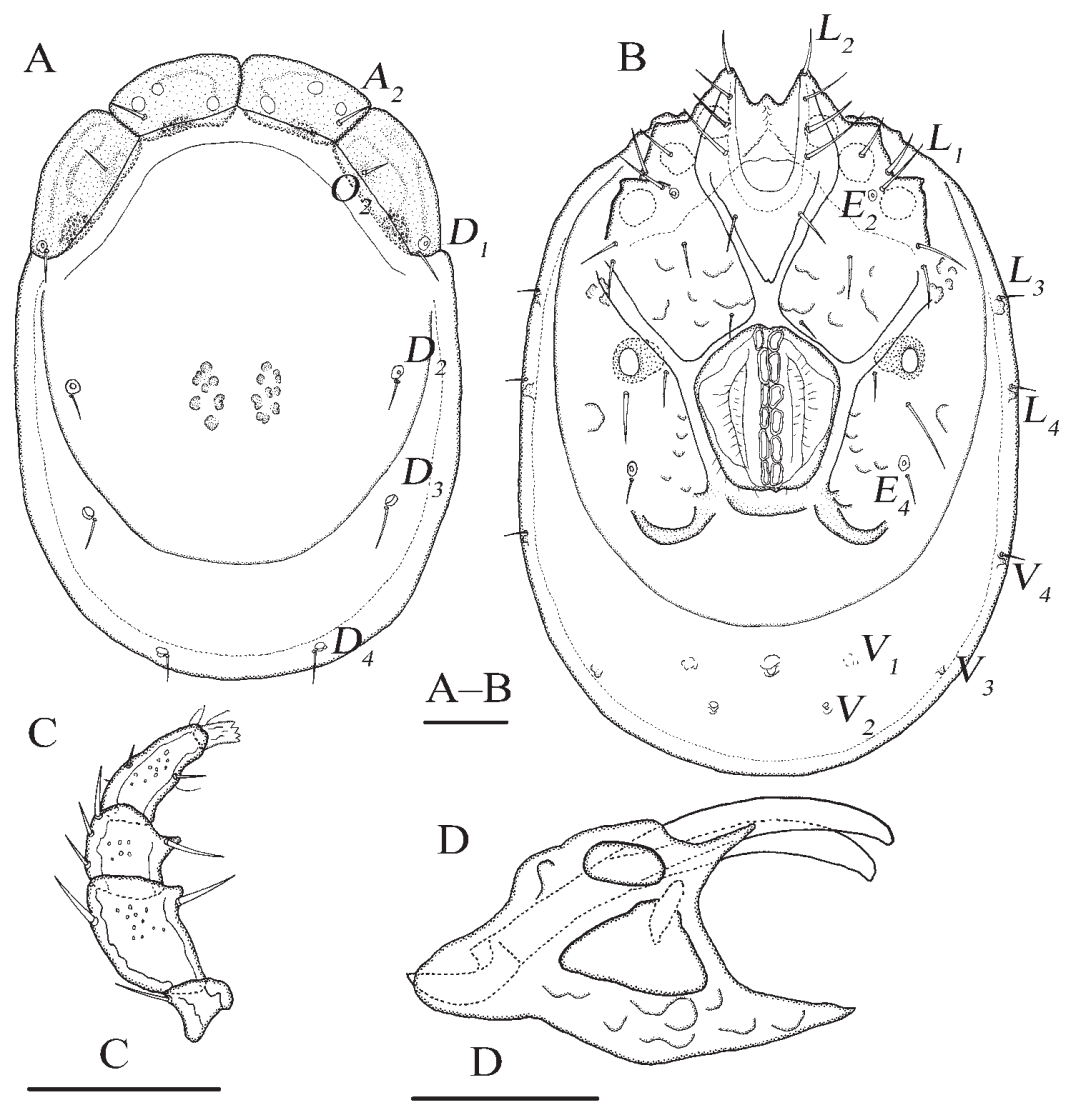
*Torrenticola pseudosiamis* Gu & Guo, sp. nov., female **A** dorsal view **B** ventral view **C** palp, lateral view **D** infracapitulum and chelicera. Scale bars: 100 μm.

**Figure 2.**
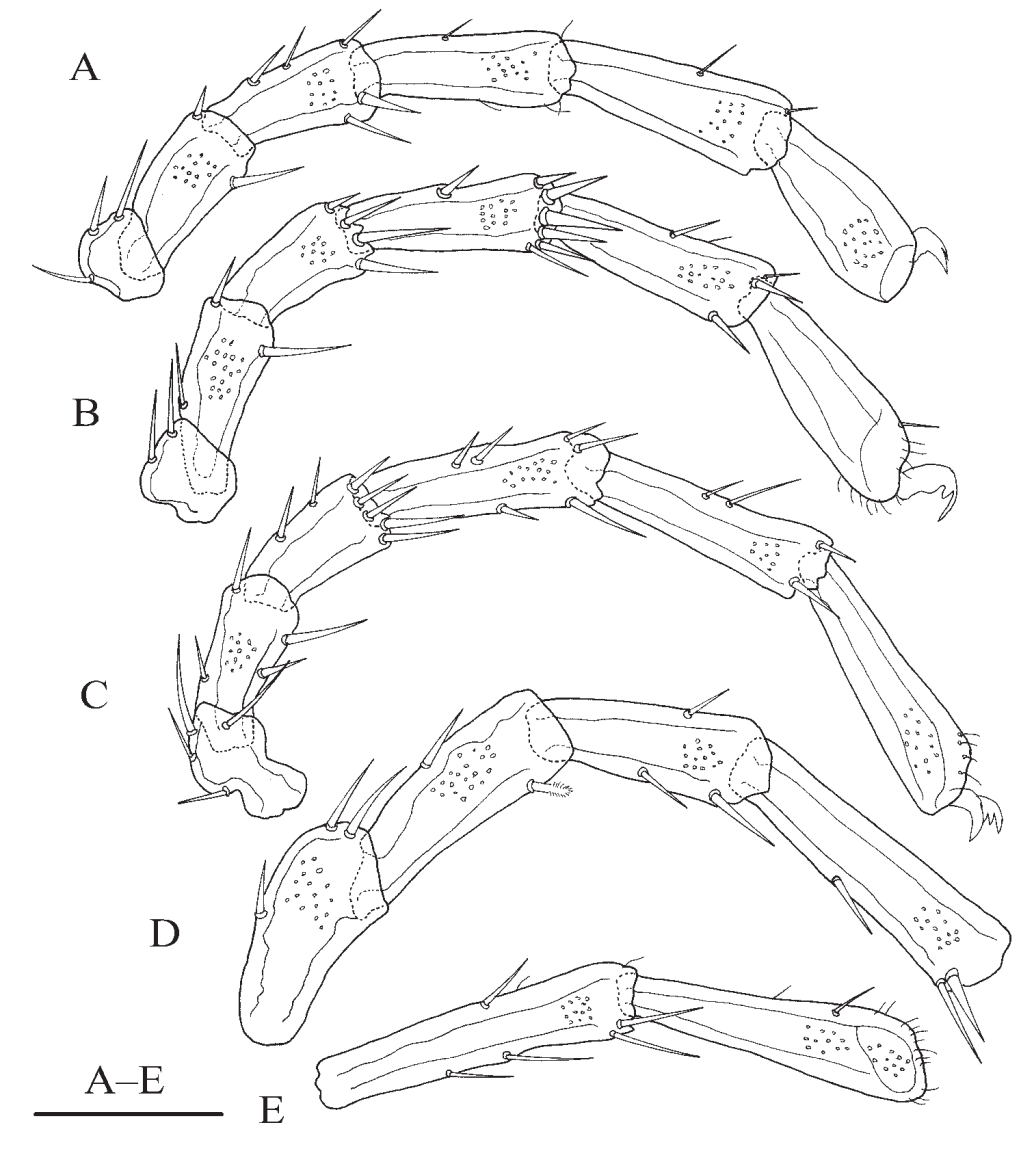
*Torrenticola pseudosiamis* Gu & Guo, sp. nov., female **A** leg-I **B** leg-II **C** leg-III **D** leg-IV-1–4 **E** leg-IV-5–6. Scale bar: 100 μm.

**Female (*n* = 3)**: Idiosoma elliptical, L 847 (774–887), W 586 (586–648), L/W ratio 1.4 (1.3–1.4). Dorsal plate 4+1 (Fig. 1A), dorsal shield L 755 (682–770), W 530 (530–585), dorsal plate L 694 (620–709), frontal platelets L 158 (152–170), W 83 (69–90), shoulder platelets L 169 (169–196), W 99 (81–99). Infracapitular bay U-shaped, depth 126 (126–150); only the tip of Cx-I exceeding to the anterior margin of idiosoma; Cx-I L 270 (230–273), mL 123 (102–123), Cx-II+III mL 33 (19–33); Gf pentagonal, L 220 (180–220), W 168 (168–192), L/W ratio 1.3 (1.1–1.3), distance between Gf and Ap 220 (156–220); *E_4_* at the same level as the 6^th^ pair of acetabula; Ap away from the line of primary sclerotization, on the same level of *V_1_* and anterior to *V_2_* (Fig. 1B). Gnathosoma (Fig. 1D): the lateral view of gnathosoma regular triangle-like; vL 231 (200–242), dL 151 (128–159), chelicera bs L 214 (202–251), claw L 41 (41–43). P-1 with one dorsal seta; P-2 with one dorsal and one ventral setae at the base of the ventral extension; P-3 with three dorsal setae and one long seta at the base of the ventral extension; P-4 with one dorsal seta in proximal half, two mediodistal setae and two ventral setae at the slight ventral extension (Fig. 1C). dL of palp segments: P-1, 27 (26–30); P-2, 68 (65–68); P-3, 41 (37–48); P-4, 62 (59–72); P-5, 20 (19–25). Legs (Fig. 2): dL of leg segments: I-L-1–6: 64 (64–74), 86 (86–87), 84 (82–92), 109 (105–123), 124 (116–124), 125 (115–125); II-L-1–6: 51 (44–51), 92 (89–116), 78 (76–86), 110 (110–132), 129 (129–149), 132 (127–142); III-L-1–6: 73 (54–73), 80 (80–108), 82 (72–86), 123 (105–123), 139 (107–139), 146 (121–146); IV-L-1–6: 126 (115–134), 119 (111–123), 118 (112–133), 169 (157–185), 169 (165–192), 167 (148–174).

**Male.** Unknown.

###### Habitat.

Streamlet.

###### Remarks.

Due to the characteristic shape of gnathosoma and dorsal shield (i.e., gnathosoma with a short rostrum, the lateral view of gnathosoma regular triangle-like), this new species is similar to *Torrenticola siamis* Pešić & Smit, 2009 (Pešić and Smit 2009). However, there are obvious differences between them: (1) only the tip of Cx-I exceeding to the anterior margin of idiosoma in this new species, but the tip of Cx-I and Cx-II exceeding in *T. siamis*; (2) *E_4_* at the same level as the 6^th^ pair of acetabula in the new species, but the 4^th^ pair in *T. siamis*; (3) *D_2_* on the same level with muscle scars in the new species, but *D_2_* anterior to muscle scars in *T. siamis*.

###### Etymology.

The specific name is from Latin affix: “*pseudo*-”, which means fake or simulated; this new species is named after its similar species, *T. siamis*.

###### Distribution.

China (Zhejiang).

##### Torrenticola anziensis

Taxon classificationAnimaliaTrombidiformesTorrenticolidae

Gu & Guo sp. nov.

A677171E-416A-59C3-AA12-7F56487AF754

http://zoobank.org/2BB83E40-0117-4BD6-8205-AF3A754EBD6B

Figures 3 , 4

###### Material examined.

***Holotype***: ♀ (SC-TO-20160701), Anzihe, Chongzhou, Sichuan Province, P. R. China (30°47'43"N, 103°12'36"E, 1690 m a.s.l.), collected by Zhuhui Ding, 29-VII-2016. ***Paratype***: 1 ♀ (SC-TO-20160702), same data as holotype.

###### Diagnosis.

Dorsal plate 4+1; infracapitular bay U-shaped and wide; genital flaps with six pairs of setae at the margins; *E_4_* at the same level as the 3^rd^ pair of acetabula; *V_1_* fused with the line of primary sclerotization; gnathosoma: rostrum long, dorsal apodemes short and sharp, ventral apodemes slender and sharp, claw short.

###### Description.

**Figure 3.**
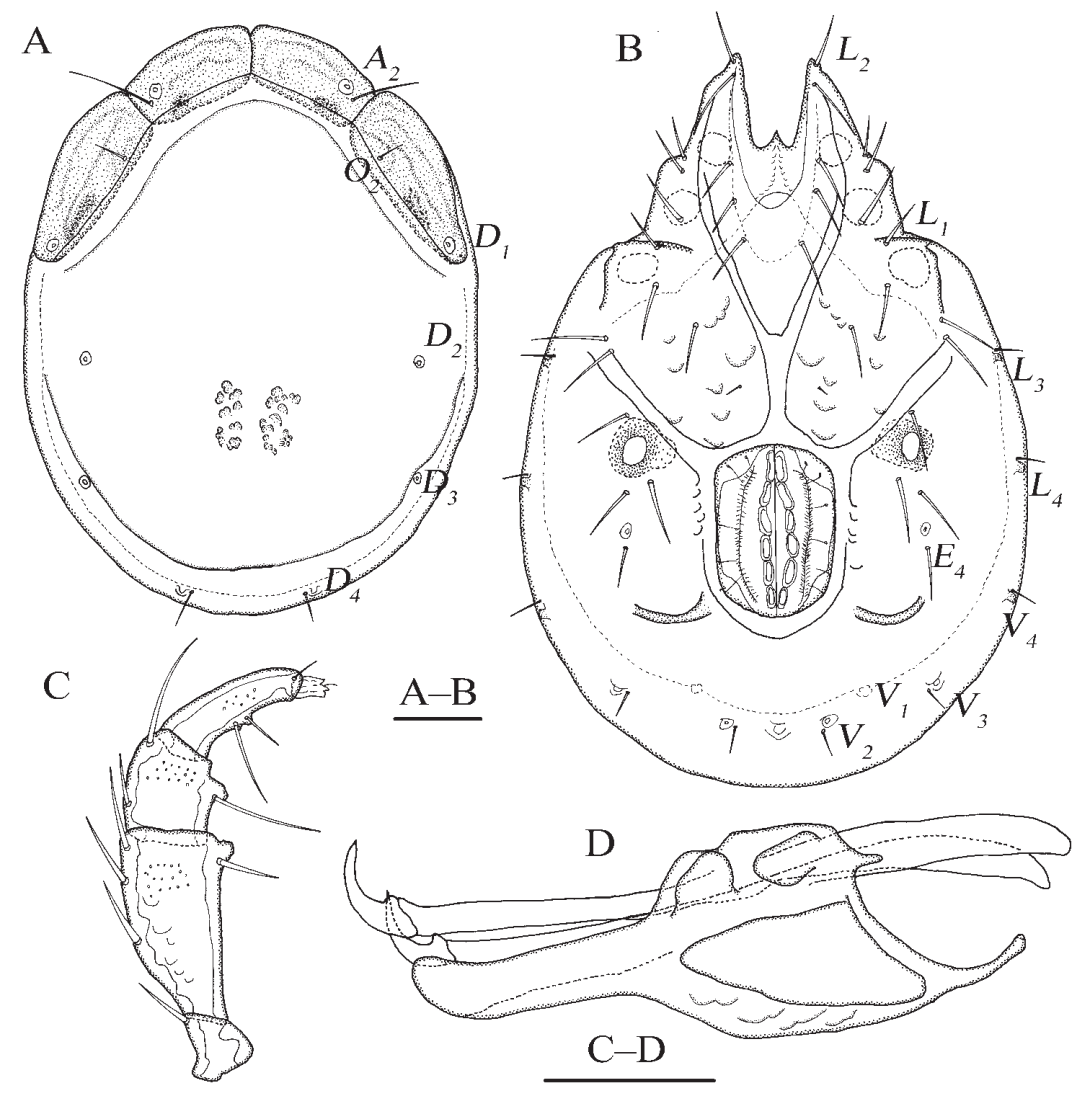
*Torrenticola anziensis* Gu & Guo, sp. nov., female **A** dorsal view **B** ventral view **C** palp, lateral view **D** infracapitulum and chelicera. Scale bars: 100 μm.

**Figure 4.**
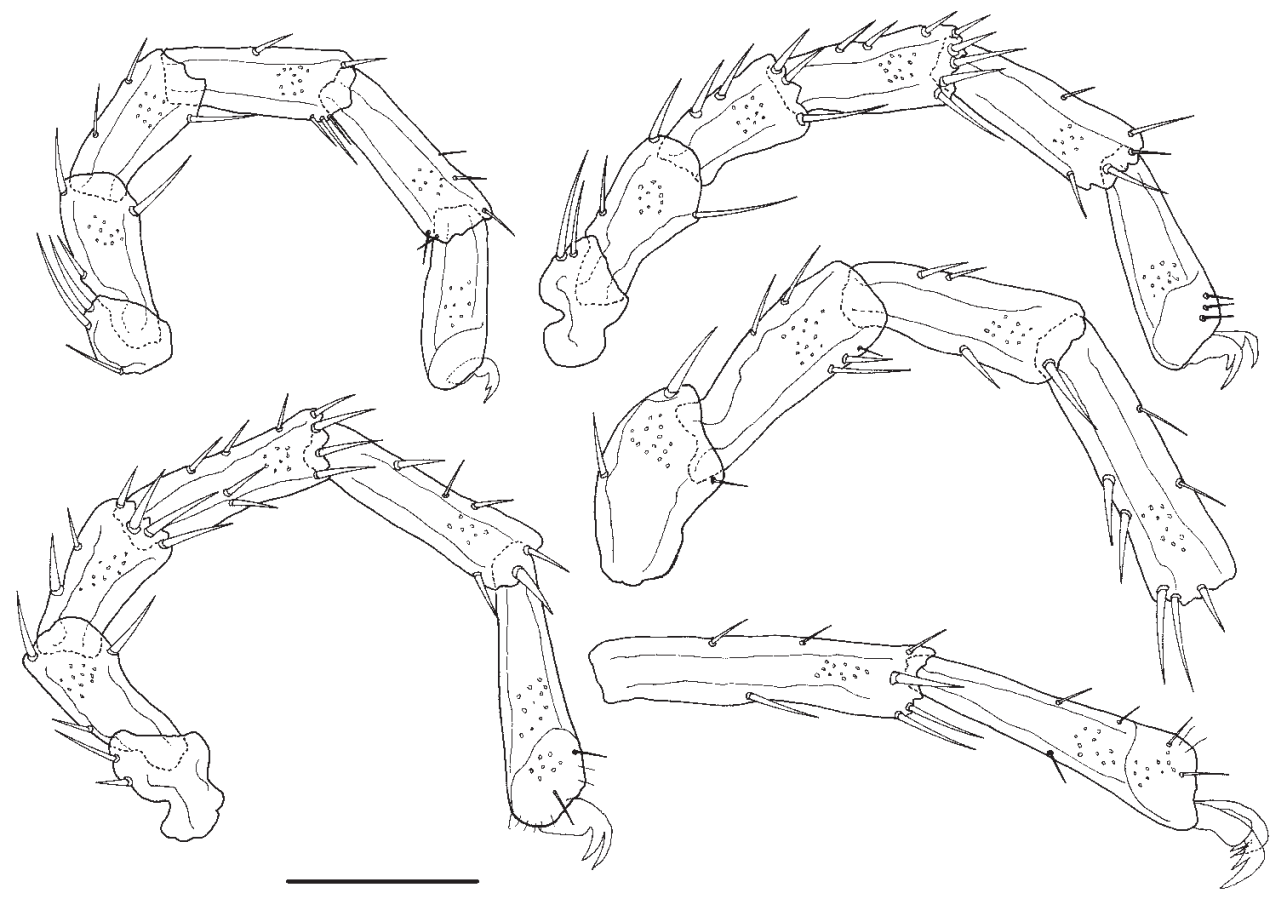
*Torrenticola anziensis* Gu & Guo, sp. nov., female **A** leg-I **B** leg-II **C** leg-III **D** leg-IV-1–4 **E** leg-IV-5, 6. Scale bar: 100 μm.

**Female (*n* = 2)**: Idiosoma elliptical, L 840 (836), W 583 (576), L/W ratio 1.4 (1.5). Dorsal plate 4+1 (Fig. 3A), dorsal shield L 680 (668), W 520 (505), dorsal plate L 617 (602), frontal platelets L 162 (158), W 78 (76), shoulder platelets L 220 (218), W 87 (89). Infracapitular bay U-shaped and wide, depth 175 (162); Cx-I L 317 (315), mL 83 (81), Cx-II+III mL 99 (89); Gf L 200 (208), W 140 (144), L/W ratio 1.4 (1.4), genital flaps with six pairs of setae at the margins; distance between Gf and Ap 123 (134); *E_4_* at the same level as the 3^rd^ pair of acetabula; Ap on the same line with *V_2_*, *V_1_* fused with the line of primary sclerotization, and *V_1_* anterior to *V_2_* (Fig. 3B). Gnathosoma (Fig. 3D): rostrum long, dorsal apodemes short and sharp, ventral apodemes slender and sharp, claw short; vL 355 (336), dL 256 (242); chelicera bs L 378 (369), claw L 63 (58). P-1 with one long dorsal seta; P-2 with three dorsal setae and one ventral seta at the base of the ventral extension; P-3 with two dorsal setae and one long seta at the base of the ventral extension; P-4 with one mediodistal seta and two ventral setae at two ventral extensions (Fig. 3C). dL of palp segments: P-1, 42 (39); P-2, 113 (109); P-3, 67 (66); P-4, 93 (95); P-5, 18 (16). Legs (Fig. 4): dL of leg segments: I-L-1–6: 44 (39), 95 (102), 88 (85), 102 (108), 110 (116), 96 (101); II-L-1–6: 43 (45), 98 (102), 93 (94), 121 (118), 136 (129), 149 (139); III-L-1–6: 43 (45), 98 (102), 81 (85), 101 (108), 117 (115), 114 (121); IV-L-1–6: 107 (102), 118 (115), 125 (119), 161 (158), 179 (183), 166 (171).

**Male.** Unknown.

###### Habitat.

Streamlet.

###### Remarks.

Due to the characteristic shape of gnathosoma and dorsal shield (i.e., gnathosoma with a long rostrum, dorsal and ventral apodemes sharp), this new species is similar to *Torrenticola haliki* Pešić & Smit, 2010 (Pešić and Smit 2010). However, there are obvious differences between them: (1) ventral projection of P-2 nose-shaped in *T. haliki*, but normally shaped in this new species; (2) P-4 with one long and three short setae at the base of ventral extensions and one thick mediodistal seta in *T. haliki*, but only with two ventral setae and one fine mediodistal seta in this new species; (3) ratio Cx-I mL/Cx-II+III mL 1.2 (male), 1.7–2.3 (female) in *T. haliki*, but 0.8 (female) in this new species.

###### Etymology.

This new species is named after Anzi (Anzi River), where the new species was collected.

###### Distribution.

China (Sichuan).

#### Genus *Monatractides* (K. Viets, 1926)

##### Monatractides sichuanensis

Taxon classificationAnimaliaTrombidiformesTorrenticolidae

Gu & Guo sp. nov.

225CB29B-8954-5683-B54A-E43DE50DD47A

http://zoobank.org/8EFE370B-7D4D-46B4-9D87-2608AE618101

Figures 5 , 6

###### Material examined.

***Holotype***: ♀ (SC-TO-20160703), Anzihe National Nature Reserve, Chongzhou, Sichuan Province, P. R. China (30°47'43"N, 103°12'36"E, 1690 m a.s.l.), collected by Zhuhui Ding, 29-VII-2016. ***Paratype***: 1 ♀ (SC-TO-20160704), same data as holotype.

###### Diagnosis.

Infracapitular bay U-shaped and wide; the tip of Cx-I with a papillary cuticular extension, *E_4_* at the same level as the posterior margins of Gf; *V_1_* fused with the line of primary sclerotization; *D_2_* on the same level with muscle scars; dorsal and ventral apodemes of gnathosoma slender and sharp; dorsal seta on P-1 long.

###### Description.

**Figure 5.**
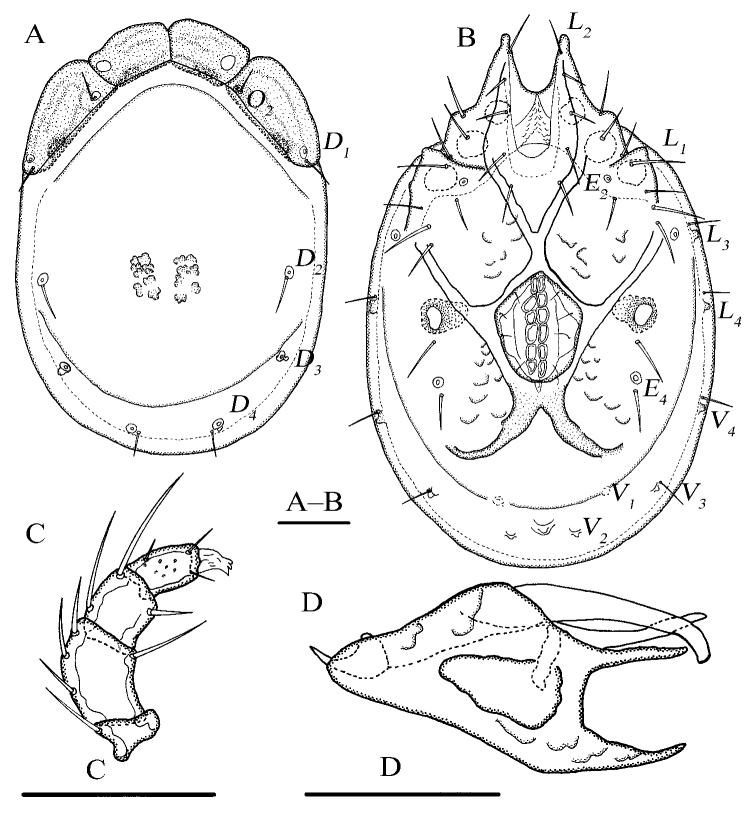
*Monatractides sichuanensis* Gu & Guo, sp. nov., female **A** dorsal view **B** ventral view **C** palp, lateral view **D** infracapitulum and chelicera. Scale bars: 100 μm.

**Figure 6.**
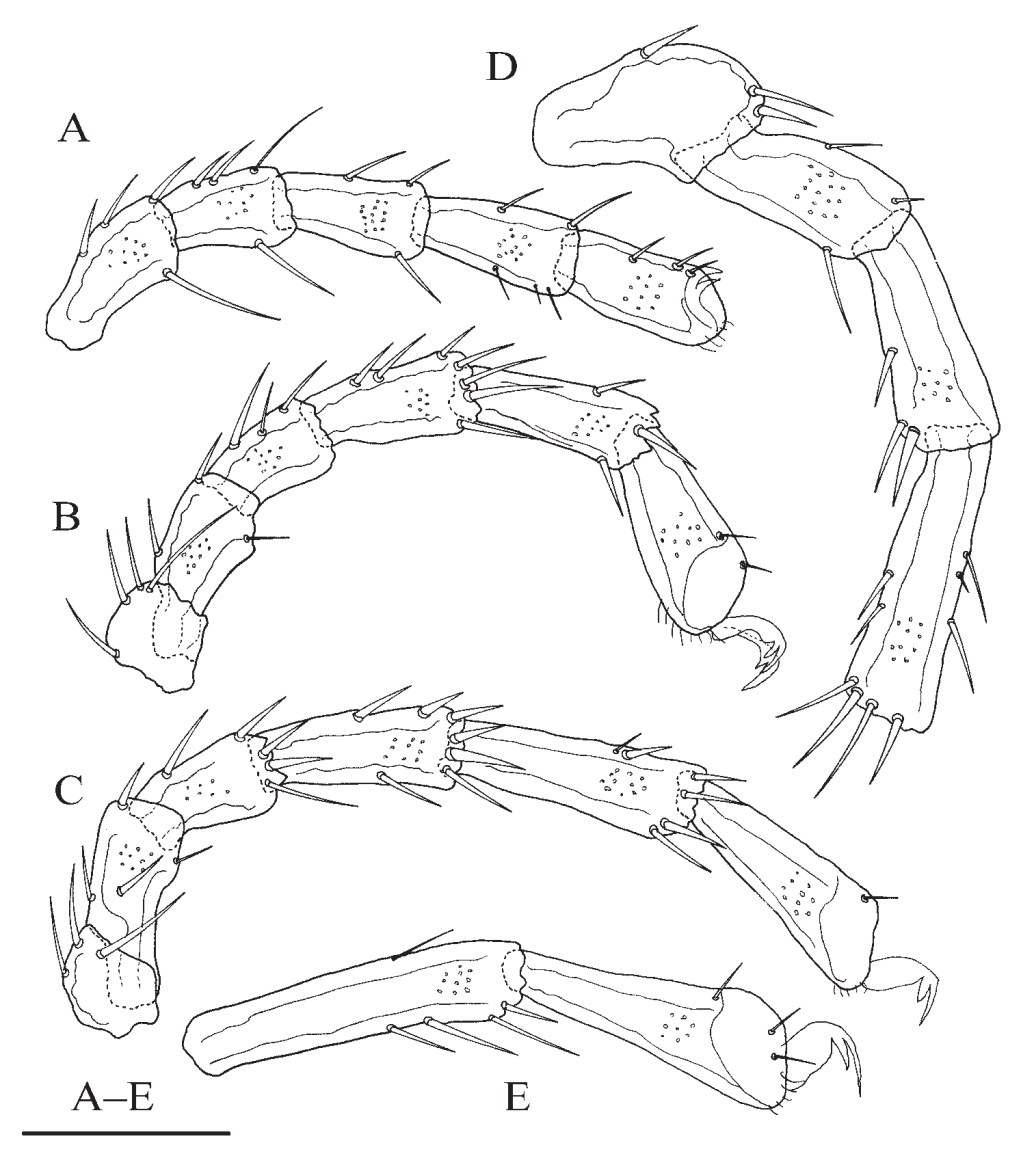
*Monatractides sichuanensis* Gu & Guo, sp. nov., female **A** leg-I **B** leg-II **C** leg-III **D** leg-IV-1–4 **E** leg-IV-5, 6. Scale bar: 100 μm.

**Female (*n* = 2)**: Idiosoma elliptical, L 749 (715), W 497 (469), L/W ratio 1.5 (1.5). Dorsal plate 4+1 (Fig. 5A), dorsal shield L 628 (586), W 438 (536), dorsal plate L 584 (423), frontal platelets L 118 (120), W 57 (68), shoulder platelets L 174 (157), W 80 (75). Infracapitular bay U-shaped and wide, depth 171 (163); the margins of Cx-II and Cx-III blunt and flat, the tip of Cx-I with a papillary cuticular extension, Cx-I L 292 (266), mL 123 (102), Cx-II+III mL 46 (31); Gf pentagonal, L 156 (159), W 122 (109), L/W ratio 1.3 (1.5), genital flaps with six pairs of setae at the margins; distance between Gf and Ap 222 (181); *E_4_* at the same level as the posterior margins of Gf; *V_1_* fused with the line of primary sclerotization, Ap at the same line with *V_2_* (Fig. 5B). Gnathosoma (Fig. 5D) vL 182 (177), dL 177 (116); dorsal and ventral apodemes slender and sharp; claw short, chelicera bs L 159 (162), claw L 27 (22). P-1 with one long dorsal seta; P-2 with three dorsal and one ventral setae; P-3 with two long dorsal setae and one short ventral seta; P-4 with one short dorsal seta and two mediodistal setae (Fig. 5C). dL of palp segments: P-1, 21 (25); P-2, 52 (48); P-3, 34 (32); P-4, 34 (39); P-5, 17 (14). Legs (Fig. 6): dL of leg segments: I-L-1–6: 57 (46), 70 (71), 59 (62), 67 (80), 58 (72), 79 (83); II-L-1–6: 55 (61), 92 (85), 56 (60), 86 (75), 84 (92), 108 (96); III-L-1–6: 63 (51), 99 (92), 71 (69), 94 (95), 118 (112), 120 (115); IV-L-1–6: 113 (115), 97 (87), 115 (111), 138 (140), 151 (126), 144 (142).

**Male.** Unknown.

###### Habitat.

Streamlet.

###### Remarks.

This new species is similar to *Monatractides harveyi* Pešić & Smit, 2012 (Pešić and Smit 2012) in the general shape of gnathosoma, but differs in: (1) P-4 with small denticles near the insertion of the ventral setae in *M. harveyi*, but smooth in the new species; (2) the margins of Cx-II and Cx-III sharp in *M. harveyi*, but blunt and flat in the new species; (3) *D_2_* anterior to muscle scars in *M. harveyi*, but *D_2_* on the same level with muscle scars in the new species.

###### Etymology.

This new species is named after Sichuan Province, where it was collected.

###### Distribution.

China (Sichuan).

##### Monatractides macrocorpis

Taxon classificationAnimaliaTrombidiformesTorrenticolidae

Gu & Guo, 2019

82581258-93B6-5DBD-AA47-83BC97F5EDC6

Figures 7 , 8

###### Material examined.

Qingliangfeng National Nature Reserve, Lin’an, Zhejiang Province, P. R. China (30°6'44"N, 118°53'36"E, 940 m a.s.l.), collected by Xinyao Gu, 31-VII-2018, 1 ♀(ZJ-TO-20180704), 2 ♂♂ (ZJ-TO-20180705, ZJ-TO-20180706).

###### Morphology.

**Figure 7.**
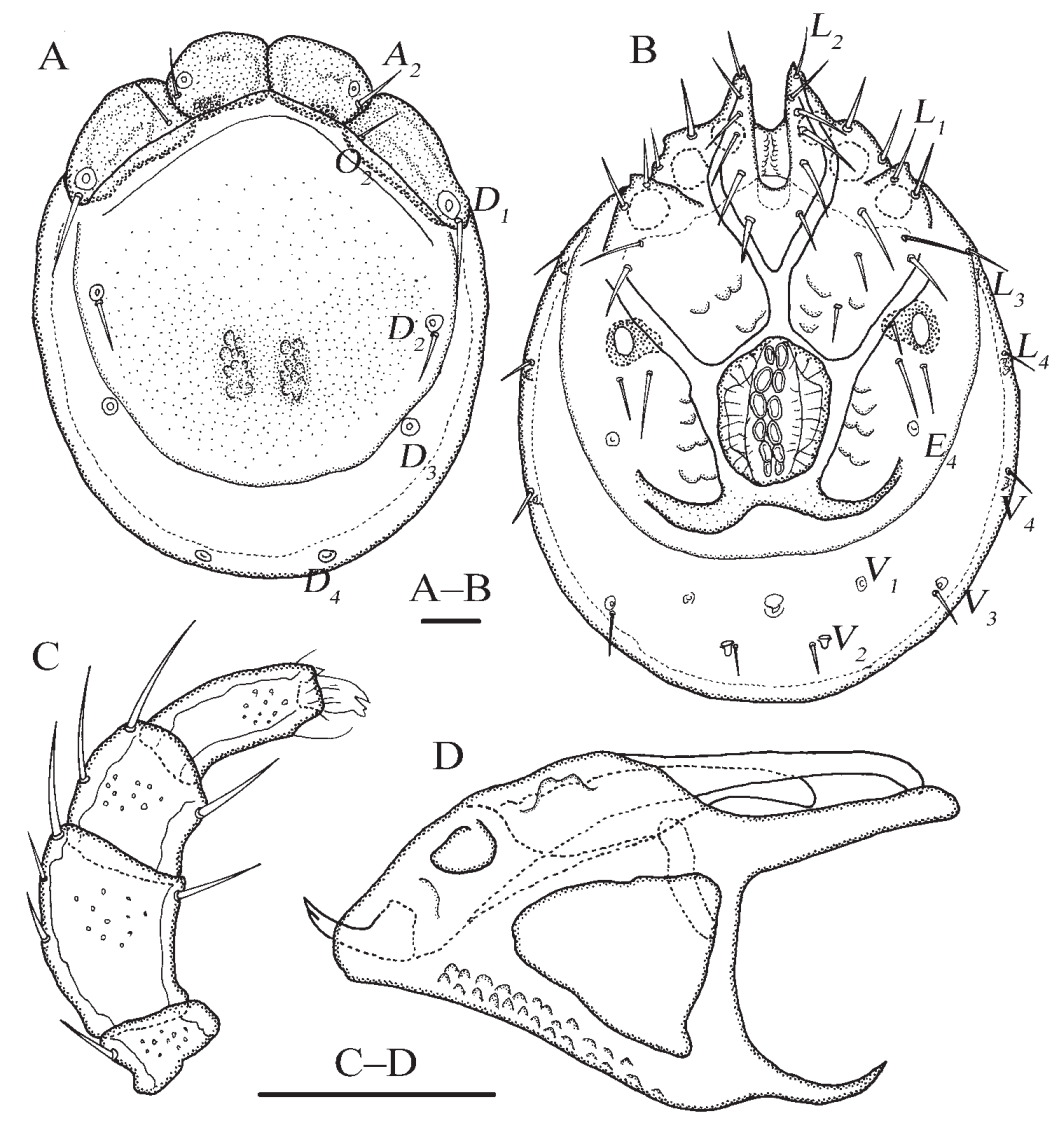
*Monatractides macrocorpis* Gu & Guo, 2019, male **A** dorsal view **B** ventral view **C** palp, lateral view **D** infracapitulum and chelicera. Scale bars: 100 μm.

**Male (*n* = 1)**: Idiosoma L 1083, W 833, L/W ratio 1.3. Dorsal plate 4+1 (Fig. 7A) with a red colour patterns, dorsal shield L 898, W 749, dorsal plate L 804, frontal platelets L 181, W 103, shoulder platelets L 254, W 115. Infracapitular bay U-shaped, depth 206; Cx-I L 376, mL 177, Cx-II+III mL 65; Gf elongated and oval, L 246, W 188, L/W ratio 1.3; distance between Gf and Ap 206. Gnathosoma (Fig. 7D) vL 231, dL 158; dorsal apodeme blunted and ventral apodeme sharp; chelicera bs L 284, claw L 27. P-1 with one dorsal seta; P-2 with three dorsal and one ventral setae; P-3 with two dorsal and one long ventral setae; P-4 with one ventral seta on the slight ventral extension (Fig. 7C). dL of palp segments: P-1, 34; P-2, 80; P-3, 54; P-4, 74; P-5, 30. Legs: dL of leg segments: I-L-1–6: 70, 129, 114, 147, 144, 125; II-L-1–6: 70, 116, 107, 151, 188, 164; III-L-1–6: 86, 146, 108, 169, 216, 192; IV-L-1–6: 152, 159, 176, 220, 241, 224. Ejaculatory complex: L 326, aL 221.

**Figure 8.**
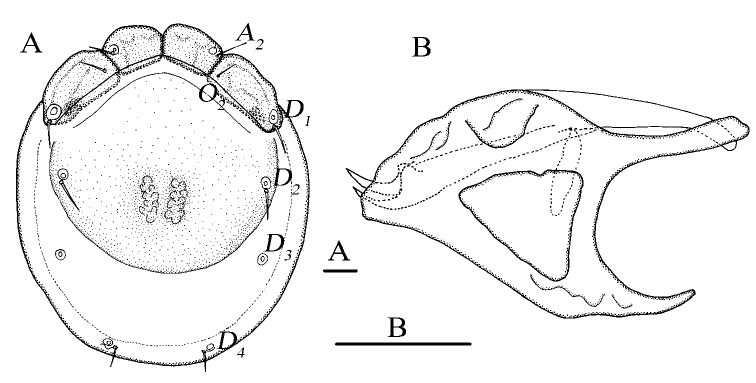
*Monatractides macrocorpis* Gu & Guo, 2019, female **A** dorsal view **B** infracapitulum and chelicera. Scale bars: 100 μm.

**Female (*n* = 2).** Body features same as the male except: Idiosoma L 1212 (1213), W 945 (949), L/W ratio 1.2 (1.3). Dorsal plate (Fig. 8A), dorsal shield L 959 (989), W 871 (845), dorsal plate L 918 (928), frontal platelets L 195 (173), W 109 (96), shoulder platelets L 269 (295), W 123 (117). Infracapitular bay depth 220 (222); Cx-I L 369 (402), mL 145 (176), Cx-II+III mL 68 (38); Gf L 256 (267), W 253 (236), L/W ratio 1.0 (1.1); distance between Gf and Ap 292 (248). Gnathosoma (Fig. 8B) vL 257 (259), dL 246 (262), claw short L 32 (29), chelicera bs L 246 (262). dL of palp segments: P-1, 32 (34); P-2, 90 (91); P-3, 51 (58); P-4, 77 (83); P-5, 21 (32). Legs: dL of leg segments: I-L-1–6: 100 (77), 114 (137), 121 (120), 153 (157), 147 (149), 127 (123); II-L-1–6: 90 (78), 134 (148), 99 (98), 154 (175), 195 (203), 168 (176); III-L-1–6: 101 (-), 161 (170), 119 (120), 177 (193), 214 (228), 217 (203); IV-L-1–6: 144 (159), 181 (160), 188 (192), 235 (243), 245 (261), 230 (220).

###### Habitat.

Streamlet.

###### Remarks.

The populations from Zhejiang Province fit the definition of *Monatractides macrocorpis* Gu & Guo, 2019 (Gu et al. 2019b). Differences with the original description are: (1) the ventral apodeme of gnathosoma, sharp in Zhejiang specimens, but blunt in Hunan specimens; (2) with a red colour pattern in Zhejiang specimens, but purple in Hunan specimens.

###### Distribution.

China (Hunan, Zhejiang).

##### Monatractides xiaoxiensis

Taxon classificationAnimaliaTrombidiformesTorrenticolidae

Gu & Guo, 2019

57566E15-04FD-502A-B9BF-847E1350D36E

Figure 9

###### Material examined.

Qingliangfeng National Nature Reserve, Lin’an, Zhejiang Province, P. R. China (30°6'12"N, 118°55'12"E, 440 m a.s.l.), female, collected by Xinyao Gu, 31-VII-2018, 3 ♀♀ (ZJ-TO-20180707 – ZJ-TO-20180709).

###### Morphology.

**Figure 9.**
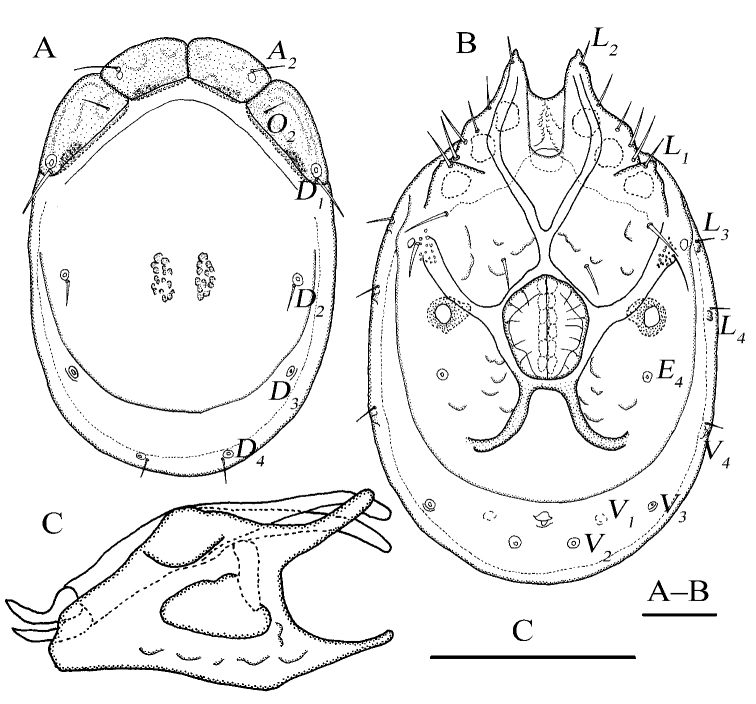
*Monatractides xiaoxiensis* Gu & Guo, 2019, female **A** dorsal view **B** ventral view **C** infracapitulum and chelicera. Scale bars: 100 μm.

**Female (*n* = 3)**: Idiosoma elliptical, L 741 (722–762), W 473 (472–491), L/W ratio 1.6 (1.5–1.6). Dorsal plate 4+1 (Fig. 9A), dorsal shield L 631 (595–656), W 428 (413–441), dorsal plate L 567 (541–579), frontal platelets trapezoidal, L 131 (120–138), W 62 (62–74), shoulder platelets triangular, L 155 (142–155), W 76 (72–79). Infracapitular bay depth 134 (134–150); Cx-I L 269 (244–269), mL 133 (92–133), Cx-II+III mL 37 (37–42); Gf L 132 (132–151), W 116 (115–118), L/W ratio 1.1 (1.1–1.3), distance between Gf and Ap 197 (197–217); Ap away from the line of primary sclerotization, on the same line with *V_1_* and anterior to *V_2_* (Fig. 9B). Gnathosoma (Fig. 9C) vL 152 (152–173), dL 117 (113–121); claw short L 23 (19–23), chelicera bs L 159 (159–172). dL of palp segments: P-1, 23 (20–23); P-2, 52 (43–52); P-3, 34 (34–38); P-4, 44 (43–44); P-5, 15 (15–19). dL of leg segments: I-L-1–6: 66 (60–66), 71 (71–81), 76 (67–76), 97 (90–98), 97 (94–97), 92 (88–94); II-L-1–6: 63 (58–63), 80 (72–85), 68 (60–76), 93 (88–93), 107 (96–107), 109 (107–115); III-L-1–6: 54 (54–58), 93 (87–93), 71 (71–74), 99 (97–109), 127 (114–127), 117 (112–127); IV-L-1–6: 102 (99–114), 94 (88–108), 115 (109–118), 145 (133–154), 154 (151–156), 147 (135–149).

**Male.** Unknown.

###### Habitat.

Streamlet.

###### Remarks.

The specimens match the general morphology of *Monatractides xiaoxiensis* Gu & Guo, 2019, a species from China (Gu et al. 2019b). *Monatractides xiaoxiensis* is characterized by: frontal platelets trapezoidal, shoulder platelets triangular; only the tip of Cx-I and Cx-II exceeding to the anterior margin of idiosoma; tips of Cx-I with an elongated cuticle extension; gnathosoma dorsal apodeme long with a blunt end, ventral apodeme pointed and bent towards dorsum; bs curved heavily towards ventrum (Gu et al. 2019b). According to these characters, we believe our specimens from Zhejiang Province are *M. xiaoxiensis*. The only differences are found in *V_4_*, which is away from the line of primary sclerotization in Zhejiang specimens, but close to the line in Hunan specimens.

###### Distribution.

China (Hunan, Zhejiang).

## Supplementary Material

XML Treatment for Torrenticola pseudosiamis

XML Treatment for Torrenticola anziensis

XML Treatment for Monatractides sichuanensis

XML Treatment for Monatractides macrocorpis

XML Treatment for Monatractides xiaoxiensis

## References

[B1] Ding ZH Guo JJ Yi TC Jin DC (2019) New water mites of the genus *Neumania* (Acari, Hydrachnidia: Unionicolidae) from China. Systematic and Applied Acarology 24(1): 1–15. 10.11158/saa.24.1.1

[B2] Goldschmidt T (2007) Studies on Latin American water mites of the genus *Torrenticola* Piersig, 1896 (Torrenticolidae, Hydrachnidia, Acari). Zoological Journal of the Linnean Society 150: 443–678. 10.1111/j.1096-3642.2007.00305.x

[B3] Gu XY Guo JJ (2019) Five new species of genera *Torrenticola* and *Monatractides* (Acari, Hydrachnidia, Torrenticolidae) from Hainan Island, China. Systematic and Applied Acarology 24(12): 2460–2482. 10.11158/saa.24.12.12

[B4] Gu XY Jin DC Yi TC Guo JJ (2019a) Contributions to the knowledge of torrenticolid water mites (Acari: Hydrachnidia) in Doupengshan, China. Zootaxa 4695(2): 101–121. 10.11646/zootaxa.4695.2.1 31719352

[B5] Gu XY Jin DC Yi TC Guo JJ (2019b) Taxonomic notes on genus *Monatractides* K. Viets 1926 (Acari, Hydrachnidia, Torrenticolidae) from China. International Journal of Acarology 45(5): 293–306. 10.1080/01647954.2019.1622593

[B6] Gu XY Jin DC Guo JJ (2020a) Three new species and one new record of Torrenticolidae (Acari, Hydrachnidia) from Wuyishan with an updated key for Chinese fauna. European Journal of Taxonomy 625: 1–23. 10.5852/ejt.2020.625

[B7] Gu XY Jia L Jin DC Guo JJ (2020b) Four new species of Torrenticola (Acari, Hydrachnidia, Torrenticolidae) from northeastern China. Zootaxa 4779(2): 245–259. 10.11646/zootaxa.4779.2.6 33055790

[B8] Gu XY Jia L Jin DC Guo JJ (2020c) New water mites of Torrenticolidae (Acari, Hydrachnidia) from Jiangxi Province, P. R. China. Acarologia 60(2): 488–500. 10.24349/acarologia/20204381

[B9] Jin DC (1997) Hydrachnellae-Morphology Systematics a Primary Study of Chinese Fauna. Guizhou Science and Technology Publishing House, Guiyang, 356 pp. [in Chinese]

[B10] Pešić V Smit H (2009) Water mites of the family Torrenticolidae Piersig, 1902 (Acari: Hydrachnidia) from Thailand, Part I. The genera *Torrenticola* Piersig, 1896, *Neoatractides* Lundblad, 1941 and *Pseudotorrenticola* Walter, 1906. Zootaxa 1982: 38–62. 10.11646/zootaxa.1982.1.2

[B11] Pešić V Smit H (2010) New records of water mites (Acari: Hydrachnidia) from Malaysia, with descriptions of three new species. Zootaxa 2354(1): 19–34. 10.11646/zootaxa.2354.1.2

[B12] Pešić V Smit H (2012) Water mites of the genus *Monatractides* (Acari: Hydrachnidia, Torrenticolidae) from Australia, with descriptions of four new species. Zootaxa 3248(1): 1–24. 10.11646/zootaxa.3248.1.1

[B13] Wiles PR (1997) Asian and Oriental Torrenticolidae Piersig, 1902 (Acari: Hydrachnidia: Lebertioidea): a revision of the family and description of new species of *Torrenticola* Piersig and *Pseudotorrenticola* Walter, from Southeast Asia. Journal of Natural History 31: 191–236. 10.1080/00222939700770121

[B14] Zhang ZQ (2018) Repositories for mite and tick specimens: acronyms and their nomenclature. Systematic and Applied Acarology 23(12): 2432–2446. 10.11158/saa.23.12.12

